# The Heart of the Alzheimer's: A Mindful View of Heart Disease

**DOI:** 10.3389/fphys.2020.625974

**Published:** 2021-01-27

**Authors:** Alessandro Evangelisti, Helen Butler, Federica del Monte

**Affiliations:** ^1^Stanford Cardiovascular Institute, Stanford University, Stanford, CA, United States; ^2^School of Medicine, Department of Molecular and Cellular Biology and Pathobiology, Medical University of South Carolina, Charleston, SC, United States; ^3^Department of Medicine, Medical University of South Carolina, Charleston, SC, United States

**Keywords:** Alzheimer's disease, heart failure, proteotoxicity, protein quality control (PQC), amyloid

## Abstract

**Purpose of Review:** This review summarizes the current evidence for the involvement of proteotoxicity and protein quality control systems defects in diseases of the central nervous and cardiovascular systems. Specifically, it presents the commonalities between the pathophysiology of protein misfolding diseases in the heart and the brain.

**Recent Findings:** The involvement of protein homeostasis dysfunction has been for long time investigated and accepted as one of the leading pathophysiological causes of neurodegenerative diseases. In cardiovascular diseases instead the mechanistic focus had been on the primary role of Ca^2+^ dishomeostasis, myofilament dysfunction as well as extracellular fibrosis, whereas no attention was given to misfolding of proteins as a pathogenetic mechanism. Instead, in the recent years, several contributions have shown protein aggregates in failing hearts similar to the ones found in the brain and increasing evidence have highlighted the crucial importance that proteotoxicity exerts via pre-amyloidogenic species in cardiovascular diseases as well as the prominent role of the cellular response to misfolded protein accumulation. As a result, proteotoxicity, unfolding protein response (UPR), and ubiquitin-proteasome system (UPS) have recently been investigated as potential key pathogenic pathways and therapeutic targets for heart disease.

**Summary:** Overall, the current knowledge summarized in this review describes how the misfolding process in the brain parallels in the heart. Understanding the folding and unfolding mechanisms involved early through studies in the heart will provide new knowledge for neurodegenerative proteinopathies and may prepare the stage for targeted and personalized interventions.

## Introduction

Heart failure (HF) and Alzheimer's disease (AD) are age-dependent diseases that affect millions of people with rapidly increasing incidence. The lifetime risk of developing HF from 45 to 95 years of age is high (20–45%) and the number of death attributable to HF is also substantially high (Benjamin et al., 2019). Meanwhile, the growth prevalence of AD from 65 to 85 years of age is 3–32% and one in three seniors dies with AD or other dementias (2020). Importantly, HF and AD share risk factors in addition to age, which include epidemiological profiles, genetic predisposition, redox characteristics, and protein homeostasis (Ames et al., 1993; Willis and Patterson, 2013; Huang et al., 2016; Benjamin et al., 2019). While dementia has been known to co-occur in patients with HF (1977; Cohen and Mather, 2007; De Toledo Ferraz Alves et al., 2010; Cermakova et al., 2015), evidence showed, only recently, that HF may co-occur in patients with AD (Troncone et al., 2016) with an estimate prevalence of about one third of cases (Reitz et al., 2007).

Key pathological hallmark of AD is the accumulation of protein aggregates as plaques, composed mainly of amyloid beta (Aß), and tangles mostly composed of hyperphosphorylated tau protein, featuring a defect in the folding of proteins and/or failure to correct or clear them. While protein homeostasis (proteostasis) has long been known to play a crucial role in the development of numerous neurodegenerative diseases such as AD, Parkinson's, and Huntington's disease, only recently, the importance of proteostasis has been recognized also for numerous diseases of various organs, including the heart. Those are now clustered as “proteinopathies.”

Proteostatic failure has been shown in several cardiac pathologies, including hypertrophic, dilated and ischemic cardiomyopathies, atrial fibrillation and cardiac amyloidosis (Gorza and Del Monte, 2005; Predmore et al., 2010; Willis and Patterson, 2013; Del Monte and Agnetti, 2014; Rabbani and Thornalley, 2019).

**Figure 1:**
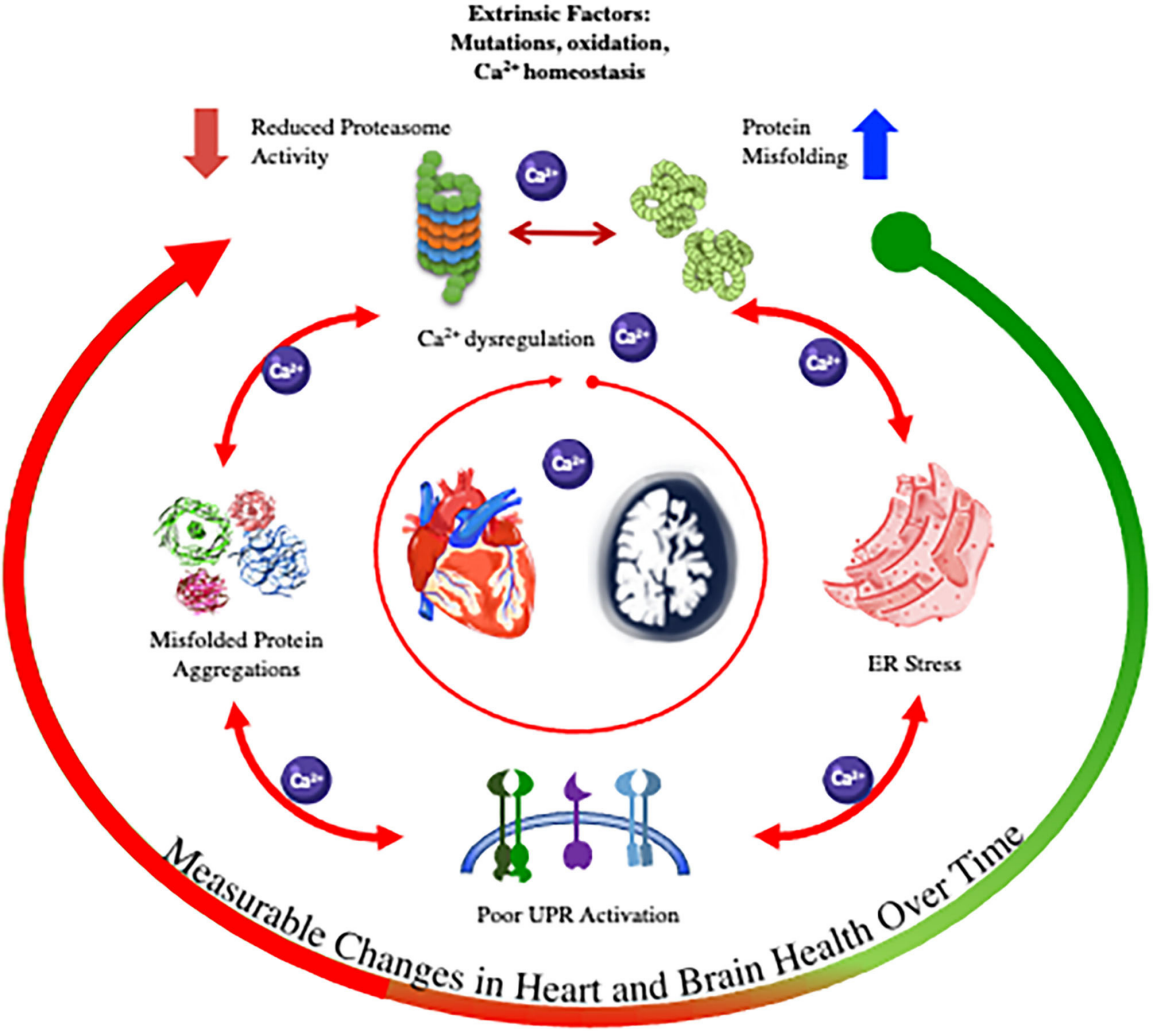
Graphic abstract illustrating the pathways of proteinopathies in the heart and brain.

Thus, in this review we summarize the processes of protein folding and misfolding and present the commonalities between the pathophysiology of protein misfolding diseases in the heart and the central nervous system (CNS) (summarized in the graphic abstract in Table 1, Figure 1). We will then focus on the direct link between cardiomyopathy and AD and how this finding is changing the current view of cardiac amyloidosis.

**Table 1:** Summary and references for the major findings described in the review.

**Significant feature**	**Key findings**	**References**
A*β* Aggregates in Heart and Brain	Plaque-like amyloid deposits in patients diagnosed with idiopathic dilated cardiomyopathy (iDCM)	Gianni et al., 2010; Troncone et al., 2016; Tublin et al., 2019
Ca^2+^ Hypothesis	Reduced proteasome activity leads to Ca^2+^ dysregulation which favors protein misfolding	Uddin et al., 2020
	Intracellular Ca^2+^ homeostasis disruption promotes cell dysfunction and death	Demuro et al., 2005, 2010; Gianni et al., 2010; Yamasaki-Mann et al., 2010; Torres et al., 2011; Demuro and Parker, 2013
ER Stress in Heart and Brain	ER stress increases in failing hearts	Okada et al., 2004; Guan et al., 2011; Park et al., 2012; Castillero et al., 2015; Uddin et al., 2018, 2020
	Inhibition of ER stress due to Aβ overload inhibits neurol apoptosis in AD mice	He et al., 2020
	Physical exercise alleviates cognitive impairment in AD mouse by reducing the expression of abnormal ER stress markers	Hong et al., 2020
Enhancing UPR^ER^ and UPR^mt^ to treat neurodegenerative and cardiovascular disease	ATF6 reprograms proteostasis, induces MANF, a protein folding chaperone in the heart, and downregulates the levels of β-site APP cleaving enzyme in AD models.	Blackwood et al., 2019a; Arrieta et al., 2020; Du et al., 2020
	GRP78, an ER Ca^2+^ dependent chaperone, protects against ischemic myocardial injury and restore ER stress in AD models	Vitadello et al., 2003; Pan et al., 2004; Shintani-Ishida et al., 2006
	Overexpression of LonP1, one of the main mitochondrial proteases, can mitigate cardiac injury during ischemic-reperfusion	Venkatesh et al., 2019
	Enhancement of UPR^mt^ in pressure-overloaded mice hearts significantly improves cardiomyocyte survival and and cardiac contractile function	Smyrnias et al., 2019
Targeting UPS to treat neurodegenerative and cardiovascular disease	LMP-2, a proteasome subunit, allows the breakdown of pre-existing sarcomeres, required for cardiac remodeling processes.	Petersen et al., 2020
	Triad3A, a E3 ligase, attenuates cardiac hypertrophy	Lu et al., 2020
	Resveratrol (RES) blocks immunoproteasome subunits (β1i, β2i and β5i) and reduces cardiac hypertrophy	Chen et al., 2019; Xie et al., 2020; Yan et al., 2020
	cAMP/PKA enhances clearance of aggregation-prone proteins in neurodegenerative diseases	Lin et al., 2013; Ranek et al., 2013; Lokireddy et al., 2015; Myeku et al., 2016
	cGMP/PKG positively regulates proteasome-mediated proteolysis degrading mutated CryAB in desmin cardiomyopathy	Liu et al., 2006a,b; Ranek et al., 2013
Enhancing autophagy to treat neurodegenerative and cardiovascular disease	TRAF6/p62 expression may relieve Aβ-mediated inhibition of p75 ^NTR^ polyubiquitination and restore neuronal cell survival	Babu et al., 2005; Geetha et al., 2012; Chen et al., 2020

## Aggregates: Indexes of Misfolding—Origin and Consequences in the Heart and Brain

Naturally, each protein undergoes a rigorous folding process to acquire its specific (native) conformation, which ultimately dictates the role and the localization of the protein itself (Hartl, 2011). Although folding of proteins is a necessary step for their function, it represents an energetically unfavorable process requiring a crew of facilitator proteins (participating in the protein quality control—PQC—described below) to timely and correctly achieve protein's native conformation. Failure to fold occurs normally, but is accelerated and amplified under conditions of cellular stress or pathological plights leading to misfold of the proteins from their stable and functional conformations and their precipitation as amyloid aggregates.

Amyloid aggregates are formed from misfolded globular, soluble proteins as a result of a combination of various factors including: a constant increase in the concentration of the precursor proteins with/out an acquired or hereditary mutation, an intrinsic propensity to misfold, or a proteolytic remodeling of a wild-type protein into an amyloidogenic fragment (Merlini, 2017; Picken, 2020). Following these processes, misfolded proteins progressively aggregate as soluble mono to poly-mers, protofibrils and ultimately as mature insoluble fibrils.

As misfolded proteins become hydrophobic, insoluble, and very resistant to degradation during the aggregation process, they also become highly reactive and unstable, leading to cellular stress and death and, ultimately, organ dysfunction (Falk et al., 2016; Merlini, 2017; Picken, 2020). Amply reported within different organ systems, pre-fibrillar species such as the soluble pre-amyloid oligomers (PAO) (Cleary et al., 2005; Demuro et al., 2010; Gianni et al., 2010; Del Monte and Agnetti, 2014 and protofibrils exert a potent toxic effect, known as proteotoxicity, driving, or contributing to the progression of the disease (Liao et al., 2001; Shi et al., 2010; Merlini, 2017; Picken, 2020). PAO and other pre-amyloidogenic species have been found to correlate with disease severity independently of amyloid fibril formation (Balducci et al., 2010; Currais et al., 2016; Chen and Mobley, 2019). For instance, a study by Diociaiuti et al., have shown that globular dimers, trimers and tetramers were the most toxic species, while monomers and larger aggregates such as insoluble amyloid plaques are almost inert in cultured primary hippocampal neurons (Diociaiuti et al., 2014; Mclendon and Robbins, 2015).

The mechanism of proteotoxicity was first identified by Demuro who demonstrated that the application of Aß PAO on neuroblastoma cells rapidly induces intracellular Ca^2+^ release, whereas the increase was not detectable when comparable amounts of monomeric and fibrillar forms were used (Demuro et al., 2005). PAO induced-Ca^2+^ release leading to cytosolic and organelles Ca^2+^ overload would in turn worsen the folding of proteins by inducing ER and oxidative stress, ultimately activating several pathogenic and apoptotic pathways. These findings lead to the introduction of the “Ca^2+^ hypothesis” of AD (Demuro et al., 2005, 2010; Yamasaki-Mann et al., 2010; Demuro and Parker, 2013) appending the “amyloid hypothesis” that wanted the accumulation of misfolded proteins as the primary driver of AD pathogenesis.

Contrary to the brain disease, Ca^2+^ dishomeastasis has been long recognized as key to the pathogenesis of myocardial dysfunction and HF (Gwathmey and Morgan, 1985; Gwathmey et al., 1987; Bers, 2002; Bers et al., 2003). Instead, only in the recent years, protein misfolding has been introduced as a novel pathogenic mechanism of cardiomyopathy. As in the brain, Aß PAO were found in the myocardium of patients diagnosed with idiopathic dilated cardiomyopathy (iDCM) and in the heart of AD patients (Sanbe et al., 2004; Gianni et al., 2010; Troncone et al., 2016). Paralleling the findings in neurons, PAO promote an increase in cytosolic and decrease in sarcoplasmic reticulum (SR) Ca^2+^ in cardiomyocytes, causing cell dysfunction and death (Gianni et al., 2010) adding a new possible cause of Ca^2+^ dishomeastasis in the failing heart. SR is a specialized ER in cardiomyocytes. Among other functions, SR tightly regulates the storage and fine-tuned control of Ca^2+^ spatial distribution and concentrations for the various Ca^2+^-dependent cellular functions. Those include the cyclic rise and fall of Ca^2+^ that drives cardiomyocytes contractility (Bers, 2002; Glembotski, 2012) as well as the stable SR Ca^2+^ content required for the proper function of the Ca^2+^ dependent chaperones (GRP78 and 94) and of protein folding. Thus, changes in Ca^2+^ homeostasis and compartmentalization in the SR and Ca^2+^ dysfunction would induce not only the known abnormal cellular contractile function but also misfolding of protein and activation of the cellular response to the unfolded proteins accumulation (UPR) (Torres et al., 2011). Reduced SR Ca^2+^ content, commonly found in failing cardiomyocytes, would induce UPR activation, as demonstrated in AC16 cells treated with SERCA2a inhibitors (Castillero et al., 2015). Those findings highlight the delicate balance required for Ca^2+^ homeostasis in cardiomyocytes and provide a novel mechanism at the origin of the well-known Ca^2+^ dishomeastasis in HF.

In addition to cause intracellular Ca^2+^ dysregulation, *per se* activating the apoptotic pathways, proteotoxicity lead to cellular dysfunction and death through other mechanisms (Ruz et al., 2020). For instance, PAO-induced toxicity can cause disruption of mitochondrial membrane integrity and ultimately lead to metabolic cellular dysfunctions (Mclendon and Robbins, 2015). Moreover, it has been shown that proteotoxicity itself can activate a broad spectrum of pro-inflammatory pathways and lead to cell death independently of amyloid plaque formation (Wright et al., 2013; Currais et al., 2016).

Finally, accumulation of amyloid aggregate can also result from failure of the PQC, i.e., the unfolded protein response (UPR) and the ubiquitin proteasome system (UPS). Those can become overloaded with misfolded proteins or can present with reduced capacity from normal aging or malfunction (Wang et al., 2008; Predmore et al., 2010; Powell et al., 2012; Picken, 2020).

## Folding Oversee: The Protein Quality Control (PQC)

To remedy the natural course of these deleterious processes, the cell has developed molecular defense mechanisms (the PQC), which help preventing or correcting the damage and minimize the level of toxic misfolded proteins (Welch, 2004; Glembotski et al., 2019; Wang and Wang, 2020). The PQC efficiently works through the interplay between specialized folding assistant proteins in the different cellular compartments (cytosolic heat shock response—HSR; ER and mitochondrial UPR—UPR^ER^ and UPR^mt^) and the protein degradation system (Wang et al., 2013).

Due to the limited regenerative capacity of the cells of the central nervous and cardiovascular systems (neurons and cardiomyocytes) (Benfey and Aguayo, 1982; Eschenhagen et al., 2017), the proteolytic control mechanisms and protein turnover are key to their function and survival. In addition, in the heart, proteins of the contractile machinery, sarcomeric proteins, need to be continuously exchanged and remodeled in order to adapt to stressors and uninterrupted shear, making proper protein turnover of sarcomeres essential for cardiac function (Willis et al., 2008). Therefore, not surprisingly, dysfunction of the proteolytic systems and proteotoxicity are pathogenic mechanisms for neurodegenerative diseases and, now, HF (Brenner et al., 2004; Shi et al., 2010; Guan et al., 2014).

While the link between the UPR and degenerative disease of the heart and brain is now established, a conundrum lies in the unclear origin of the misfolding. Do extrinsic factors (e.g., mutations, oxidation, altered Ca^2+^ homeostasis) lead to the accumulation of protein aggregates that results in ER stress and the activation of UPR and ERAD/UPS? Or are these systems primarily dysfunctional (e.g., reduced proteasome activity), which leads to misfolded protein accumulations via Ca^2+^ dysregulation and/or oxidative stress (Uddin et al., 2020).

### The Unfolded Protein Response (UPR)

First line of defense, molecular chaperones take part on the PQC pathways by associating with misfolded proteins and assisting in the proper folding or re-folding in the different subcellular compartments.

**Figure 2:**
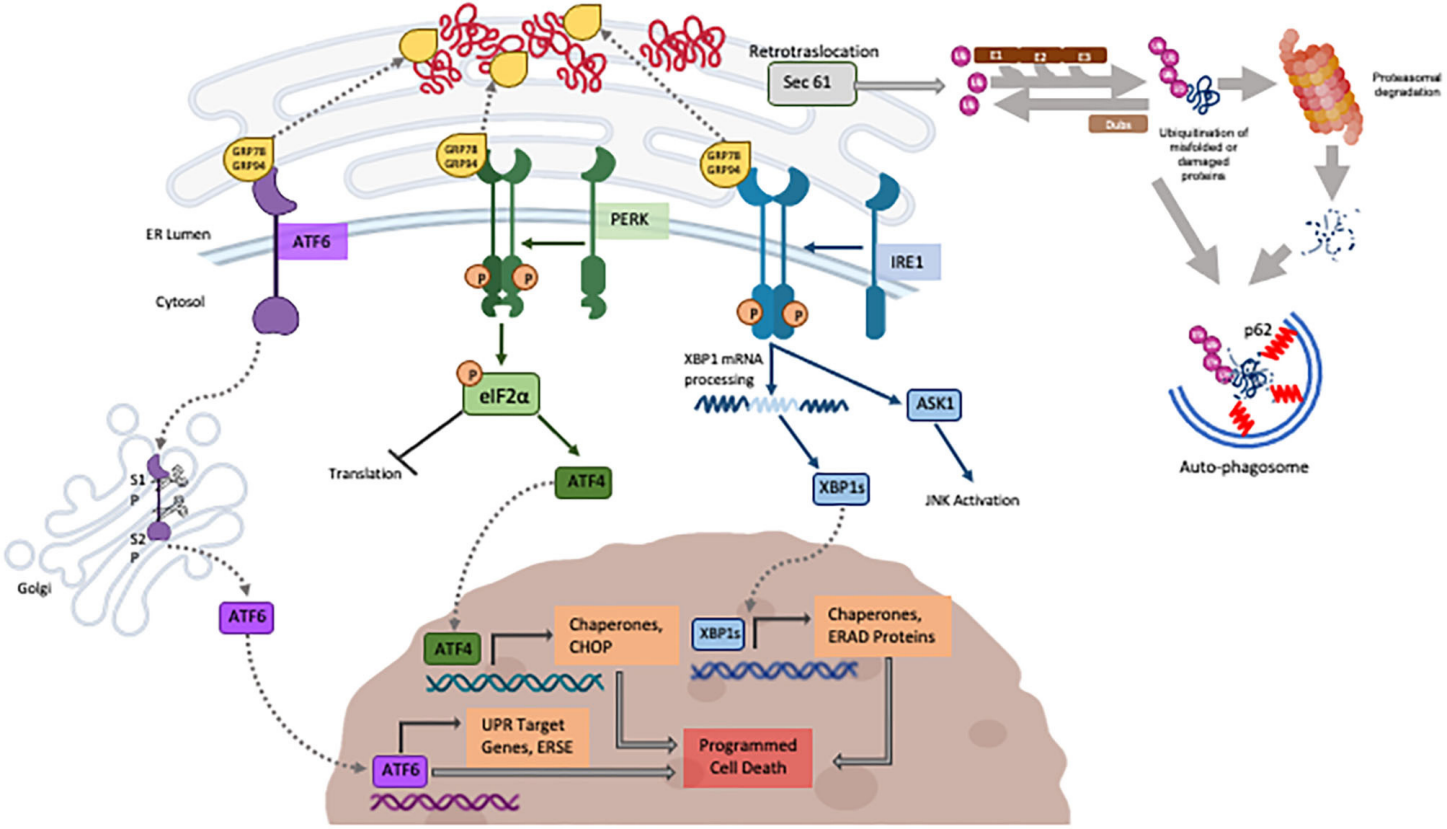
Schematic illustration of the three major known pathways composing the UPR (created with BioRender) the UPS and autophagy described in the review.

Heat shock proteins (Hsps) function to help promoting folding of cytosolic proteins and their transportation through membranes (Webster et al., 2019) while the Glucose-Regulated Proteins (GRPs), first described in mammalian cells exposed to glucose deprivation, assist the normal folding and refolding of ER/secretory proteins. Accumulation of misfolded proteins in the ER is sensed by the GRP chaperone proteins that are recruited to assist refolding while releasing the inhibition on three resident ER transmembrane sensors (PERK, IRE-1 and ATF6) (schematically illustrated in Figure 2). Those, when activated, lead to a signal cascade that triggers the refolding of proteins in the ER (Scheper et al., 2007; Glembotski, 2008; Arrieta et al., 2018) via the upregulation of the synthesis of chaperone proteins while inhibiting the overall protein synthesis to reduce the ER stress. Part of this response is the proteasome associated ER-associated degradation (ERAD), responsible for degrading terminally misfolded proteins (Plemper and Wolf, 1999). While the ER stress response is the natural reaction to cell threats and is meant to be beneficial to cells, if the ER stress is not resolved, prolonged activation of the UPR will shift to apoptotic cell death mediated by C/EBP homologous protein (CHOP), human caspase-4 and rodent caspase-12 (Wang et al., 1996; Nakagawa et al., 2000; Hitomi et al., 2004) described later. More recently discovered and less studied, the UPR^mt^ recognizes and degrades mitochondrial proteins that failed to fold. Misfolded proteins accumulated in the mitochondrial matrix are recognized by mitochondrial HSP (as HSP60) for refolding or degraded by mitochondrial proteases (as CLPP1) into peptide fragments. Those are exported to the cytosol by the HAF1 transporter enabling the inhibition of the import of the transcription factor ATFS in the mitochondria, shifting to its nuclear translocation for the transcription of protective genes for mitochondrial homeostasis (Rev in Jovaisaite et al., 2014).

### UPR in the Brain and Heart

#### HSR

The first regulators of the cytosolic UPR are the heat-shock proteins Hsps. Hsps like Hsp28, HspB8, and αB-crystallin have been found upregulated in the brain of AD patients and co-localize with Aß plaques (Shinohara et al., 1993; Renkawek et al., 1994; Wilhelmus et al., 2006). Mouse models of AD have been used to investigate the role of Hsps in AD and found that mice, which are deficient in HspB1, are more sensitive to Aß PAO toxicity (Ojha et al., 2011) and crossing Hsp27 overexpressing mice with APPswe/PS1dE9 mice leads to mice that have fewer amyloid plaques and improved cognition (Tóth et al., 2013).

Hsps are also resident in heart tissues and have been shown to have functional roles in cardiovascular disease in humans and mice. Hsp90, Hsp22, and HspB1 have been shown to be cardioprotective from stressors like oxidative stress and ischemic damage (Kupatt et al., 2004; Islam et al., 2020; Wu et al., 2020). On the other hand, mutated αB-crystallin (CryAB(R120G) was the first cytosolic small chaperone linked to cardiomyopathy (Wang et al., 2001) and polymorphisms in HSPB7 have been identified as a risk factor for iDCM through a large SNP panel of patients from Germany and France (Stark et al., 2010). These evidences indicate both a deleterious and protective role of Hsps against amyloid aggregation in both the heart and brain, yet they do not demonstrate causality.

#### UPR^ER^

*In-vitro*, mouse and human studies point to the ER stress response (UPR) as pathogenically involved in the progression of neurodegenerative diseases. Based on finding from cultured neuron, the ER stress response has been shown to be activated by Aß in AD (Yu et al., 1999; Nakagawa et al., 2000; Ferreiro et al., 2006; Seyb et al., 2006), and inhibition of Aß-induced ER stress was found to be effective in inhibiting neuronal apoptosis in some (although not all) transgenic AD mouse models (Lee et al., 2010; He et al., 2020). Impairment of the ER stress has also been shown to be the mechanism by which physical exercise mitigates cognitive impairment accompanied by reduction of the expression of ER stress markers in AD mouse brain (Hong et al., 2020). Either as primary involvement or exhaustion of the system, UPR was found impaired in human AD brains.

In the heart, models of cardiac hypertrophy by transverse aortic constriction (Okada et al., 2004; Uddin et al., 2018) result in increased levels of ER stress markers (i.e., GRP78, p-PERK, p- eIF2α, CHOP, caspase-12), suggesting a role for the UPR in HF progression and implicating ER stress as a mechanism for cardiomyocyte apoptotic cell death (Guan et al., 2011; Park et al., 2012). In human, mRNA levels of ER chaperones and markers of the UPR (GRP78/BiP, eIF2α, and XBP1) are increased in patients with HF (Okada et al., 2004; Castillero et al., 2015) supporting the translational relevance of the mice studies. However, the reversal of the stress markers activation by ventricular unloading may suggest a compensatory rather than causative role of the activation of the UPR in human HF.

As in the brain, UPR was also shown to be effective against the accumulation of PAO in the heart (Maloyan et al., 2007). Whether secondary of causative, chemical chaperones have been tested to alleviate cardiac hypertrophy and neurodegenerative disease in mice, suggesting their potential for relieving ER stress in proteinopathies (Park et al., 2012; Mimori et al., 2019). Additionally, re-chaperone therapy has been tested for neuronopathic lysosomal diseases and cystic fibrosis, a misfolded protein disease of the lungs (Suzuki, 2014). This evidence demonstrates that targeting the UPR may not only benefit the treatment of neurodegenerative diseases, but also degenerative diseases of the heart could substantially be positively adjusted.

Various reports also defined the involvement of the individual players of the UPR^ER^ for the heart and brain diseases. *The molecular chaperone* GRP78 (BiP) is reduced in patients with familial AD due to presenilin-1 (*PSEN*-1) mutations (Katayama et al., 1999; Niwa et al., 1999). The mechanism leading to this reduction was interrogated using cell culture studies of mutant mammalian cells which showed that *PSEN*-1 mutations can decrease GRP78 expression due to disturbed IRE1 function (Katayama et al., 1999; Niwa et al., 1999) while overexpression of GRP78 in *PSEN*-1 mutant cell lines restores ER stress to that of WT cells (Katayama et al., 1999; Niwa et al., 1999). In a follow up study, upregulation of GRP78 was found to be independent of presenilin expression, and the levels of this chaperone were not significantly different in the brains of sporadic AD vs. FAD (Sato et al., 2000). However, later studies found that mutant *PSEN*-1 neurons were more susceptible to cell death via ER stress and showed increased levels of the ER stress induced apoptotic mediator CHOP in *PSEN*-1 mouse models, also suggestive of ER stress (Milhavet et al., 2002; Terro et al., 2002). This suggests that either lack of or variants of *PSEN*-1 have an effect on ER-stress levels of GRP78. On the other hand, this may be due to a secondary activation via protein aggregation rather than presenilin expression or function. Upregulation of GRP78 was found in the neurons of AD patients early in disease progression regardless of presenilin mutation, but this protein did not co-localize with neuro-fibrillary tangles (Hoozemans et al., 2005), suggesting that early activation of the UPR may precede tangle formation or that the GRP78 upregulation occurs in brain cells other than neurons. Thus, while GRP78 may not be pathogenically directly linked to presenilin mutations, it may reflect the response to ER stress and protein aggregation regardless of the initiation event.

Less studied in the heart, GRP78 is upregulated in ischemic preconditioning and contributes to the protection against hypoxic conditions *in-vitro* (Pan et al., 2004) and ischemic myocardial injury *in-vivo* (Shintani-Ishida et al., 2006) while knockout of GRP78 promotes cardiomyocyte loss, HF and death (Wang et al., 2018). Similarly, overexpression of the chaperone GRP94 reduces myocyte degradation due to Ca^2+^ overload or ischemia in cultured skeletal and cardiac muscle cell cultures (Vitadello et al., 2003). Thus, while studies in the heart are limited, the data thus far suggest a cardioprotective role for ER chaperones in the heart.

The *PERK (Protein Kinase R-like ER Kinase)* pathway, one of the three transmembrane ER sensors, is activated in the AD brain (Unterberger et al., 2006). In a study of AD patients, of all ER stress markers tested, p-PERK was the only marker that correlated with disease progression, was increased in AD cases, and was largely associated with tau pathology (Buchanan et al., 2020). Correcting the defect using small-molecule PERK inhibitors showed to have therapeutic effects against neurodegeneration (Rozpedek-Kamińska et al., 2020).

While under unstressed conditions there was no effect on left ventricular mass or function (Misra et al., 2019) nor was there an effect on cardiomyocyte size, stressing the heart by aortic constriction resulted in decreased ejection fraction, increased LV fibrosis, and enhanced cardiomyocyte apoptosis in PERK knockout mice as compared to controls (Liu et al., 2014), suggesting a cardioprotective role for PERK in the heart following injury. As in the brain, PERK was shown to be protective against overload induced HF and lung remodeling in an inducible cardiac specific PERK knockout mouse line (Liu et al., 2014).

*ATF6 (Activating Transcription Factor 6)*, a second transmembrane ER sensor, has been found to downregulate the levels of β-site APP cleaving enzyme (BACE1), rescuing amyloid pathology in an AD mouse model (Du et al., 2020). Additionally, ATF6 is activated in ischemia/reperfusion injury and has shown to be protective in the brain as in the heart (Blackwood et al., 2019a), while ATF6 deletion in cardiomyocytes abrogates this protection. The mechanism of ATF6 protection in the heart was brought back to transcriptional reprogramming of proteostasis. ATF6 induces mesencephalic astrocyte-derived neurotrophic factor (MANF), which acts as a protein folding chaperone in the heart during periods of reductive stress (Arrieta et al., 2020). Further, RHEB (Ras homolog enriched in brain) was identified as another target of ATF6 in the heart during stressful conditions and acts as an activator of mTORC1 (mammalian target of rapamycin complex 1), which induces protein synthesis. The activation of these pathways during TAC was found to underlie the hypertrophic phenotype in the heart of WT mice, but not in cardiac specific ATF6 knockout mice (Blackwood et al., 2019b) validating the protective/compensatory effect of ATF6 in the heart.

Induction of *IRE1*α *(Inositol-requiring enzyme)*, a third ER sensing pathway of the UPR, phosphorylates, and activates its downstream regulator XBP-1 (X-box-binding protein) in the AD brain, further supporting the knowledge that also this branch of the UPR may be a defense activated mechanism in AD (Salminen et al., 2009; Lee et al., 2010). XBP1 is an upstream activator of the hexosamine biosynthetic pathway (HBP), the induction of which was found to be protective in the heart. Further, hypoxia has been found to specifically activate XBP1-inducible proteins within cardiac context in an *in-vitro* rat ventricular myocyte cell cultures, but the response was not further activated during reoxygenation (Thuerauf et al., 2006). Those findings support the functional response activation of the UPR in the heart, with the lack, however, of further activation upon reoxygenation possibly indicating the switch off of the response upon resolution of the insult. On the other hand, patients with end stage HF were found to have reduced XBP1 expression (Wang et al., 2014). Whether this represents an exhaustion of the system over a chronic condition or a primary defect leading to chronic myocardial damage is currently unknown. IRE1α also regulates the ASK1 (Apoptosis signal-regulating kinase 1) pathway, which is involved in Aß-induced neuronal cell death as demonstrated by the lack of Aß-induced death in ASK1^−/−^ neurons (Kadowaki et al., 2005). A similar role for ASK1 has been shown in the heart of ASK1^−/−^ mice, which showed reduced cardiomyocyte apoptosis and improved preservation of LV function compared to wildtype (Yamaguchi et al., 2003). Further elucidating the role of ASK1 in the heart, a transgenic mouse model of inducible overexpression of ASK1 found that there was greater injury after ischemia and reperfusion compared to WT (Liu et al., 2009). Thus, induction of IRE1α and its downstream effectors can have either protective or detrimental effects on the heart as shown by the cardioprotective role of XBP1 and the induction of cardiomyocyte death regulated by ASK1.

#### UPR^mt^

In addition to the ER's own system of controlling protein homeostasis, UPR^mt^ is lately receiving a lot of attention given the organelle's key role in many metabolic functions, including ATP production and intracellular Ca^2+^ regulation (Duchen, 2004; Bereiter-Hahn et al., 2008; Munch, 2018). Function and quality of mitochondria need to be tightly controlled to ensure proper metabolic supplies and to prevent the production of reactive oxygen species (ROS) (Baker et al., 2011). Thus, mitochondrial dysfunction has been implicated in numerous diseases such as major neurodegenerative diseases, Parkinson's and Alzheimer's diseases and various cardiomyopathies (Duchen, 2004; Mclendon and Robbins, 2015). Stress conditions affecting the folding of proteins would also affect the overall cell viability by impairing mitochondrial function. In such a case, UPR^mt^ is triggered and it is activated in a very short time carrying on an acute response in order to alleviate proteostasis defects and restore cell survival by increasing the expression of mitochondrial proteases and chaperones and promoting biogenesis (Urbina-Varela et al., 2020).

Given its central role for cell metabolism, UPR^mt^ is essential for cell function and for the metabolic support in both brain and heart. On the same line of thoughts as for the other UPRs players, components of the UPR^mt^ are currently been investigated as potential novel therapeutic targets in AD. Activation of UPR^mt^ genes has been showed to be a distinct feature in both familial and sporadic AD compared to healthy individuals (Beck et al., 2016). Recently, a study carried by Sorrentino et al. showed that boosting mitochondrial proteostasis by enhancing the UPR^mt^ and mitophagy ultimately decreased Aβ aggregation, reduced amyloid plaque formation and ameliorated contextual memory in the APP/PSEN1 AD mice (Sorrentino et al., 2017).

Similarly, Venkatesh et al., recently showed that enhancing UPR^mt^ by overexpressing LonP1, one of the main mitochondrial proteases, can mitigate cardiac injury during ischemia-reperfusion by preventing oxidative damage, in part by rebalancing the OXPHOS complex subunit levels (Arrieta et al., 2019; Venkatesh et al., 2019). Additionally, pharmacological enhancement of UPR^mt^ in pressure-overloaded mouse hearts significantly improved mitochondrial respiration, cardiomyocyte survival, and cardiac contractile function (Smyrnias et al., 2019).

### Ubiquitin Protease System (UPS)

Failure of the UPR to refold damaged proteins and/or resolve the ER stress result in the activation of the degradation pathways (UPS) aimed at recycling the building blocks for new protein synthesis in a process that begins with substrate polyubiquitination followed by proteasome degradation (Li et al., 2011; Ciechanover and Kwon, 2015; Gilda and Gomes, 2017). Under normal circumstances, the UPS degrades around 90% of proteins and its reach expands to all cellular compartments including nucleus, cytoplasm, ER as well as cell membranes (Schubert et al., 2000; Pagan et al., 2013). In addition to expired or environmentally damaged old proteins, as many as 30% of newly synthesized proteins are also degraded by the proteasome within a few minutes of their synthesis due to inherent difficulties in folding properly (Schubert et al., 2000; Bucciantini et al., 2002; Pagan et al., 2013).

### UPS in the Brain and Heart

When the UPR fails, the UPS combats the accumulation of toxic protein aggregates. Therefore, it is not surprising that damage of this system would have severe consequences to the brain and heart structure and function. In both organs, failure of the proteasome activity has been described in degenerative diseases. Within the brain of AD patients, proteasome inhibition is sufficient to cause neuronal death (Keck et al., 2003). In the heart, an imbalance between ubiquitination rates and degradation of ubiquitin labeled substrates is implicated in cardiomyocyte's cell death (Kostin et al., 2003).

As misfolded proteins are central biomarkers in the pathophysiology of numerous neurodegenerative diseases, with aggregates of Aß being central to AD (Willis and Patterson, 2013), several studies have shown that deficiency in Aß clearance may be a major cause of late-onset AD. Moreover, ubiquitin proteins have been observed within the intracellular tangles typical of AD (Mori et al., 1987; Perry et al., 1987). Since then, much attention has been given to the UPS in AD because of both its main role in intracellular proteolytic pathways and its presence within AD protein aggregates (Riederer et al., 2011).

Several hypotheses have been considered to explain the causes of UPS dysfunction; in addition to inherited genetic mutations, which are responsible for familial AD, there is accumulating evidence that oxidative stress signaling may have a crucial early pathogenic role in the late-onset and sporadic AD by irreversibly modifying proteins (Riederer et al., 2011). Various protein modifications such as carbonylation and nitration are generally associated with loss of function leading to either unfolding and degradation of the damaged proteins or to the aggregation and accumulation of the latter as inclusions (Butterfield et al., 2006). Different studies showed that the accumulation of oxidized protein in AD is tightly associated with dysfunction of the 20S proteasome, which is the main player in the proteolytic activity of the UPS (Szweda et al., 2002). Members of the proteasome themselves may be altered due to oxidative stress, which can lead to protein misfolding and may ultimately influence the correct functionality of the proteolytic mechanisms. Some components of the UPS themselves may be subject to oxidation or mutations, consequently influencing misfolded protein accumulation instead of their degradation (Riederer et al., 2011). Defective proteolysis has been manly seen in the hippocampus, para-hippocampal, and middle temporal gyri, and the inferior parietal lobule of AD patients; moreover, proteolytic dysfunction has been hypothesized to cause synaptic dysregulation, which is observed early in AD since protein degradation plays a fundamental role in synaptic plasticity (Wang and Huang, 2012).

In the heart, impaired proteasome activity was first shown in hypertrophic and failing human hearts in absence of changes in proteasome protein expression suggesting that post-translational modifications are responsible for the defective proteasomal degradation capacity (Predmore et al., 2010). Recent studies have focused on the role of the ubiquitin protease system (UPS) in cardiac hypertrophy as a mechanism of cardiac remodeling, a maladaptive and unfavorable outcome that can progress into overt HF (Frey and Olson, 2003), providing a molecular mechanisms for many abnormal signaling pathways in cardiomyopathies. In fact, it has recently been shown that breakdown of pre-existing sarcomeres, required for cardiac remodeling processes is optimized by the induction of the proteasome subunit low molecular weight protein (LMP)-2 (Petersen et al., 2020). Furthermore, a key regulator of normal cardiac growth and activated by exercise and pressure overload as short term adaptive response (Shiojima et al., 2005) AKT leads to cardiac hypertrophy in mice upon sustained induction (Shiojima et al., 2005) and is negatively regulated by PTEN. This signaling pathways has been shown to be modulated by two UPS players, the enzyme that catalyzes the first step in ubiquitin conjugation, UBA1 (Shu et al., 2018) and by TRIM10 (Yang et al., 2020), an E3 ubiquitin ligase that assists in the conjugation of ubiquitin to protein substrate, both degrading PTEN leading to increase AKT and hypertrophy. Additionally, investigations of Triad3A (an E3 ligase) have shown that increased expression of this E3 ligase attenuates cardiac hypertrophy (Lu et al., 2020) through ubiquitination of toll-like receptors 4 and 9 (TLR4 7 TLR9) (Lu et al., 2020). Activation of these TLRs of the innate immune system activates AKT signaling. Notably this enzyme is also implicated in neurodegenerative diseases (Husain et al., 2017). Thus, while the UPS system is implicated in cardiac remodeling and hypertrophy, reduction or activation of AKT signaling can result from divergent manipulations of UPS depending on the pathways invested.

Myocardial hypertrophy is also modulated by the immunoproteasome, an inducible form of the constitutive proteasome. Inhibition or knockout of immunoproteasome subunits (β1i, β2i, and β5i) has been shown to attenuate cardiac hypertrophy (Chen et al., 2019; Xie et al., 2020; Yan et al., 2020) a postulated mechanism underlying the effect of resveratrol (RES) (Chen et al., 2019). RES blocks immunoproteasome activity, thus preventing PTEN degradation and AKT signaling (Xie et al., 2020; Yan et al., 2020).

The role of ubiquitination in cardiac remodeling has also been documented through the inhibition of the de-ubiquitination enzymes (DUBs). Investigations into 19S proteasome deubiquitinates showed that inhibition of these DUBs prevents the breakdown of IκBα and leads to the inactivation of NF-κB, which suppresses cardiac remodeling (Hu et al., 2018). Furthermore, an additional DUB—UCHL1—is known to be dysregulated and is involved both in neurodegenerative disorders and more recently in cardiac hypertrophy (Hu et al., 2018). UCHL1 deubiquitinates epidermal growth factor receptor (Hershberger et al., 2010), preventing its degradation and resulting in the activation of multiple downstream signaling cascades that positively regulate cardiac hypertrophy (Bi et al., 2020).

Much like hypertrophic cardiomyopathy and like in many other diseases in which protein misfolding and aggregation is the major hallmark of their pathophysiology, UPS has been shown to participate to the pathogenesis of restrictive cardiomyopathy. An example is desmin-related myopathy, which affects both skeletal and cardiac muscle ultimately developing desmin-related cardiomyopathy (DRC). Genetic mutations of desmin and of its small chaperone protein, αB-crystallin, can lead to aberrant aggregation of desmin resulting in cardiomyopathy with poor disease prognosis (Chen et al., 2005). Chen et al. found that the aberrant aggregation of these proteins impairs the UPS system before cardiac hypertrophy occurs leading to heart remodeling (Chen et al., 2005).

On the same line of research, Liu et al. showed that UPS proteolytic function in the heart is severely impaired also when a human mutant desmin is expressed in transgenic mouse hearts (Liu et al., 2006a). Like other groups, they demonstrated that the deficiency likely responsible for the pathophysiology of the disease resides in the 19S regulatory cap, which helps to channel and deliver the substrate into the proteolytic chamber of the 20S core (Chen et al., 2005; Liu et al., 2006a). The results of these studies suggest that malfunctions in the UPS may directly participate in the pathogenesis of the disease, although as a secondary event to the accumulation of misfolded proteins. In fact, in a follow up study, Liu et al. showed that upregulation of members of the heat shock proteins family significantly attenuated the aberrant aggregation of desmin demonstrating that protein aggregation is a major pathogenic pathway in which misfolded proteins cause UPS impairment and malfunction (Liu et al., 2006b).

As protein aggregates are present in dilated cardiomyopathies, a defect in the ubiquitin proteasome system is foreseeable as a consequence of overwhelming of this defense mechanism. Additionally, a primary defect of the UPS has been identified in DCM though mutations in FBXO32, encoding for an E3 ubiquitin ligase (Al-Hassnan et al., 2016), a finding validated in a mouse model where FBXO32 knockout cause DCM (Al-Hassnan et al., 2016).

Additional studies further support the notion that overactivation or dysregulation of ubiquitin mediated protein degradation has a role in the progression of DCM (Li et al., 2017; Murata et al., 2018; Spanig et al., 2019). LIM domain 7 (LMO7) gene, a protein with predicted interaction domains PDZ and LIM, has been long thought to take part in regulation of UPS pathway in cardiac tissues (Li et al., 2017). Recently, it was found that DCM patients display decreased mRNA levels of LMO7 and polymorphisms of the gene have been linked to the susceptibly and prognosis of disease (Li et al., 2017). Protein arginine methyltransferase 1 (PRMT1) is universally expressed, assists in the post-translational modification of proteins, is associated with alternative splicing, and is integral for the function of multiple tissue types (Murata et al., 2018). Deletion of this protein in juvenile mice lead to the formation of cardiac alternative splicing isoforms of four genes, including ASB2, resulting in its truncation and failure to act as the proper component in an E3 ligase complex, leading to development of DCM (Murata et al., 2018). Further support for the role of the UPS in DCM was provided by a study that characterized the UPS activity in human heart tissue from cardiomyopathy patients and found decreased percentage of ubiquitin-positive cells and cells with ubiquitin deposits in end-stage cardiac tissue (Spanig et al., 2019). Additionally, a trend for reduced protein expression of MAFbx (E3-ubiquitin-ligases muscle-atrophy-F-box), a regulator of cardiac remodeling and inhibitor of hypertrophy through ubiquitin degradation of calcineurin, was found in the DCM group (Spanig et al., 2019).

Finally, in a study of 60 human patients with HF, it was found that enhanced expression of ubiquitin in cardiomyocytes is a protective mechanism in early stages of HF, whereas the absence or decreased ubiquitin expression associates with end-stage DCM (Pawlak et al., 2019).

Given the numerous studies that have demonstrated the importance of the UPS in both neurodegenerative and cardiovascular diseases, new ways of targeting the proteasome-ubiquitin machinery have been explored. Among all the post-translation modifications that subunits of the UPS undergo in order to activate the clearance mechanism, and efficiently degrade ubiquitinated proteins, phosphorylation surely plays a central role. This seems to be accomplished by most kinases such as PKA and cGMP-dependent kinase (PKG) (Ranek et al., 2013); therefore, both molecules became ideal therapeutic targets to prime the dysregulated UPS response. A study from Ranek et al. showed that PKG positively regulates proteasome-mediated proteolysis degrading mutated CryAB (Liu et al., 2006a,b; Ranek et al., 2013).

Like in the heart, multiple studies showed that enhancing PKA in different cell lines and mouse models of neurodegenerative diseases, such as tauopathies, Huntington's and Alzheimer's disease, causes an augmented clearance of aggregation-prone proteins in both the soluble and insoluble fractions (Lin et al., 2013; Ranek et al., 2013; Lokireddy et al., 2015; Myeku et al., 2016). In addition to rescue UPS proper function, enhancing cAMP has been shown to improve cognitive and memory functions in Alzheimer's patients (Vitolo et al., 2002). Studies have demonstrated that different phosphodiesters inhibitors can augment cAMP/PKA and cGMP/PKG signaling (Verplank et al., 2019), and therefore strengthen the UPS response in mouse model of AD and cardiac amyloid proteinopathy (Sheng et al., 2017; Verplank et al., 2019).

### Autophagy

Autophagy is a highly evolutionary conserved system to degrade retired or damaged proteins and organelles and selectively degrades aggregated proteins. Even though UPS and autophagy are two distinct proteolytic systems, they interact to remove long-lived proteins and unrepairable misfolded ones (Chen et al., 2020). The link between the UPS and autophagy is mediated by p62 (Rusten and Stenmark, 2010), a receptor of autophagy substrate that is activated by phosphorylation upon proteotoxic stress due to UPS inhibition, inducing autophagy (Zheng et al., 2011; Lim et al., 2015; Sha et al., 2018; Pan et al., 2020). Given its central function, changes in p62 have been linked to diseases. Defunct autophagic systems can lead to p62 accumulation, delayed arrival of waste to the proteasome and disturbance of the UPS (Korolchuk et al., 2009).

In the AD brain p62 acts to clear misfolded proteins such as hyperphosphorylated and polyubiquitinated tau through proteasomal degradation (Babu et al., 2005), reinforcing the importance of p62's relationship with AD disease progression. Indeed, decreased expression of p62 aligns with AD progression, leading to a decline in clearance of polyubiquitinated tau and promotion of tau aggregates (Alonso et al., 1994). Furthermore, cooperation of p62 and TRAF6 inhibits Aβ-mediated neuronal death through p75 ^NTR^ activity (Geetha et al., 2012). Thus, enhancing p62 and TRAF6 expression might relieve tau aggregation and Aβ-mediated neuronal death in the AD brain.

Less studied in the heart, a feedforward loop in the activation of autophagy by proteasome inhibition has been delineated through the PPP3/calcineurin-TFEB-SQSTM1/p62 pathway (Su and Wang, 2020). Studies investigating the role of p62 in protein misfolding diseases of the heart appear limited to desmin cardiomyopathy and atherosclerosis. In a mouse model of desmin cardiomyopathy by DRC-linked mutant desmin or αB-crystallin (CryAB^R120G^) and in cultured rat neonatal cardiomyocytes expressing the mutant desmin autophagic flux both *in-vivo* and *in-vitro* was augmented together with increased mRNA and protein levels of p62 (Zheng et al., 2011). Those findings provided evidence for p62/autophagy adaptive response to the forced accumulation of misfolded proteins while p62 depletion worsened the misfolded aggregates accumulation and exacerbated cell injury supporting the protective effect of p62/autophagy for proteotoxic stress.

Finally, in the atherosclerotic disease p62-enriched aggregates are recognized as a hallmark of advancing atherosclerotic plaques due to dysfunctional autophagy (Sergin et al., 2016).

## The Direct Link Between AD and HF

With still unidentified protein precursor, the discovery of protein aggregates, in the form of both extracellular plaques and intracellular amyloid fibers, in the hearts of patients with primary diagnosis of iDCM (Gianni et al., 2010) helped to link the heart and brain proteinopathies. Subsequent characterization showed that prefibrillar myocardial deposits in iDCM were biochemically analogous to those found in AD (Subramanian et al., 2015), and later, misfolding, aggregation, and deposition of amyloid-beta (Aß) were found in the myocardium of patients with primary diagnosis of AD compromising myocardial function effectively pathogenically linked the disease in both organs (Troncone et al., 2016). Whether the link between HF and AD being of metastatic or systemic etiology is still to be determined, evidence support the possible dual nature of the link. In support of the systemic etiology genetic variants in the presenilin gene (PSEN) were identified not only in familial (Li et al., 2006), but also in sporadic (Gianni et al., 2010) cases of dilated cardiomyopathies, while the abovementioned deposits of Aß in patients with AD may also support the metastatic route of Alzheimer's cardiomyopathy—the traditional route of cardiac involvement in the SAA, AL, and TTR amyloidosis. Either ways, these findings show the heart-brain axis can be framed as an organ system that can be exploited by mechanisms (e.g., protein misfolding) that result in physiological and pathological disease in both organs.

## Conclusions

Emerging data has led to the formation of new hypotheses regarding the relationship between AD and HF and has opened novel research avenues for basic science and clinical research. The common pathogenesis of proteinopathies is supported by the accumulation of misfolded protein aggregates, the involvement of proteotoxicity and defects in protein quality control systems in disease of both the heart and the brain. The focus of future studies should highlight the unclear origin of protein misfolding in degenerative diseases of the cardiovascular and central systems. Increasing understanding of cellular and molecular mechanisms involved in the onset and progression of protein misfolding diseases will overall allow to develop more precise and targeted therapeutic approaches.

## Data Availability Statement

The original contributions presented in the study are included in the article/supplementary material, further inquiries can be directed to the corresponding author/s.

## Author Contributions

AE and HB reviewed the literature and wrote the draft. FdM edited the draft to the final version. All authors contributed to the article and approved the submitted version.

## Conflict of Interest

The authors declare that the research was conducted in the absence of any commercial or financial relationships that could be construed as a potential conflict of interest.
